# *Dictyostelium* Dynamin Superfamily GTPases Implicated in Vesicle Trafficking and Host-Pathogen Interactions

**DOI:** 10.3389/fcell.2021.731964

**Published:** 2021-10-13

**Authors:** Ana Katic, Dario Hüsler, François Letourneur, Hubert Hilbi

**Affiliations:** ^1^Institute of Medical Microbiology, University of Zürich, Zurich, Switzerland; ^2^Laboratory of Pathogen Host Interactions, Université de Montpellier, CNRS, INSERM, Montpellier, France

**Keywords:** amoebae, *Dictyostelium discoideum*, effector protein, large GTPase, host-pathogen interaction, *Legionella*, *Mycobacterium*, vesicle trafficking

## Abstract

The haploid social amoeba *Dictyostelium discoideum* is a powerful model organism to study vesicle trafficking, motility and migration, cell division, developmental processes, and host cell-pathogen interactions. Dynamin superfamily proteins (DSPs) are large GTPases, which promote membrane fission and fusion, as well as membrane-independent cellular processes. Accordingly, DSPs play crucial roles for vesicle biogenesis and transport, organelle homeostasis, cytokinesis and cell-autonomous immunity. Major progress has been made over the last years in elucidating the function and structure of mammalian DSPs. *D. discoideum* produces at least eight DSPs, which are involved in membrane dynamics and other processes. The function and structure of these large GTPases has not been fully explored, despite the elaborate genetic and cell biological tools available for *D. discoideum*. In this review, we focus on the current knowledge about mammalian and *D. discoideum* DSPs, and we advocate the use of the genetically tractable amoeba to further study the role of DSPs in cell and infection biology. Particular emphasis is put on the virulence mechanisms of the facultative intracellular bacterium *Legionella pneumophila*.

## *Dictyostelium Discoideum* as a Model to Study Membrane Dynamics and Host-Pathogen Interactions

The social soil amoeba *Dictyostelium discoideum* is a primordial phagocyte in its unicellular form and has been used as a model organism to analyze developmental processes, cytokinesis, vesicle trafficking, cell motility and migration, as well as host cell-pathogen interactions. In some instances, *D. discoideum* represents a valid model for human diseases, because the amoeba harbor a number of genes, which are implicated (in mutant form) in human diseases (Müller-Taubenberger et al., 2013). The haploid genome of *D. discoideum* is approximately 34 Mb large, comprises six chromosomes and encodes approximately 12,500 predicted proteins, including a large number of mammalian orthologs (Eichinger et al., 2005). A large *D. discoideum* mutant collection is available from the *Dictyostelium* stock center^1^ (Gaudet et al., 2011), and the amoeba can be genetically manipulated rather easily, using a remarkable repertoire of molecular genetic tools. Non-essential genes can be disrupted or replaced by homologous recombination, RNA interference has been described, and ectopic expression of endogenous or foreign genes is straightforward (Kuspa and Loomis, 1992; Faix et al., 2004; Kuhlmann et al., 2006; Paschke et al., 2018). In fact, recent cell biological and infection studies using dually labeled *D. discoideum* strains and confocal laser scanning microscopy or imaging flow cytometry revealed intriguing mechanistic insights into the virulence of the facultative intracellular bacterium *Legionella pneumophila* (Weber et al., 2014, 2018; Bärlocher et al., 2017a; Steiner et al., 2017; Welin et al., 2018; Hüsler et al., 2021).

Membrane dynamics, vesicle trafficking and organelle identity are controlled by a class of compartment-specific phospholipids termed phosphoinositide (PI) lipids (Payrastre et al., 2001; Di Paolo and De Camilli, 2006; Michell, 2008; Balla, 2013). The headgroup phosphatidylinositol (PtdIns) is reversibly mono- or polyphosphorylated by PI kinases and phosphatases at the 3′, 4′, and/or 5′ positions of the inositol ring. *D. discoideum* defined deletion mutants have been instrumental to elucidate the role of PI-metabolizing enzymes for membrane dynamics and vesicle trafficking, or cell motility and chemotaxis. Representative examples include PI 3-kinases (PI3Ks) and the PI 3-phosphatase PTEN (phosphatase and tensin homolog), which spatially and temporally regulate chemotaxis (Funamoto et al., 2002; Kölsch et al., 2008). The PI 5-phosphatase Dd5P4, a homolog of the mammalian enzyme OCRL (oculocerebrorenal syndrome of Lowe), acts on PtdIns(4,5)*P*_2_ and PtdIns(3,4,5)*P*_3_ and is implicated in phagocytosis (Loovers et al., 2007). The PI 5-kinase PIKfyve phosphorylates PtdIns(3)*P* during endocytosis (Buckley et al., 2019). Finally, RpkA (receptor phosphatidylinositol kinase A), a seven-helix transmembrane protein with a GPCR (G protein coupled receptor) signature and a predicted PtdIns(4)*P* 5-kinase domain is also implicated in phagocytosis and endocytosis (Riyahi et al., 2011).

In addition to the analysis of cell biological processes, *D. discoideum* has also been used as a model to study cellular host-pathogen interactions. In particular, the virulence of amoeba-resistant bacterial pathogens, such as *L. pneumophila* (Hoffmann et al., 2014b; Swart et al., 2018) or *Mycobacterium marinum* (Cardenal-Muñoz et al., 2017) can be assessed in *D. discoideum* at high spatial resolution and by live-cell microscopy. A drawback of using *D. discoideum* as an infection model for *L. pneumophila* is that the amoebae grow optimally at 23°C and die off beyond 26°C, while the pathogen has a growth optimum of 37°C and grows considerably slower at an infection temperature of 25°C. However, since many basic cell biological pathways are conserved in *D. discoideum* and mammalian cells, the amoebae are a powerful and versatile model to study the pathogenesis of *L. pneumophila* despite this limitation.

**FIGURE 1:**
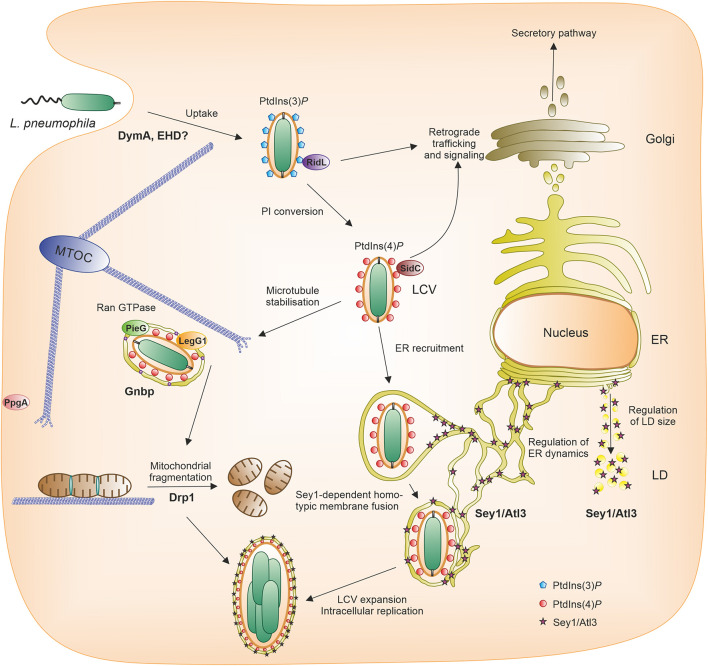
Pathogen vacuole formation and intracellular replication of *L. pneumophila*. Following uptake of *L. pneumophila* by the host cell, a unique pathogen vacuole forms, the *Legionella*-containing vacuole (LCV). The LCV restricts interactions with lysosomes, but communicates with the endosomal, secretory and retrograde trafficking pathways and finally intimately associates with the ER. A hallmark of LCV maturation is the phosphoinositide (PI) lipid conversion from the endosomal PtdIns(3)*P* to the secretory PtdIns(4)*P*, both of which serve as membrane anchors of distinct *L. pneumophila* effector proteins (e.g., RidL, SidC). The RCC1 repeat domain effectors LegG1/MitF, PieG or PpgA localize to the LCV or the plasma membrane, respectively, and activate the small GTPase Ran to stabilize microtubules. In the course of LCV maturation, large GTPases also play a role. The dynamin superfamily protein (DSP) Sey1/Atl3 catalyzes pathogen vacuole expansion, the fission DSP Drp1 promotes intracellular bacterial replication by modulating mitochondrial fusion/fission dynamics, and the scaffold DSP Gnbp localizes to the pathogen vacuole. The *D. discoideum* DSP DymA and EHD possibly play a role for bacterial uptake. Accordingly, different DSPs play a role in LCV formation either *in cis* (on the LCV) or *in trans* (in a distance from the LCV). ER, endoplasmic reticulum; LCV, *Legionella*-containing vacuole; LD, lipid droplet; MTOC, microtubule organizing center; PI, phosphoinositide.

The Gram-negative environmental bacterium *L. pneumophila* is the causative agent of a life-threatening pneumonia termed Legionnaires’ disease (Newton et al., 2010). The main virulence factor of *L. pneumophila* is the Icm/Dot type IV secretion system (T4SS), which translocates more than 300 different “effector” proteins into host cells, where they subvert pivotal processes (Finsel and Hilbi, 2015; Qiu and Luo, 2017; Swart et al., 2020a). The opportunistic pathogen replicates intracellularly within a unique compartment, the *Legionella*-containing vacuole (LCV), in free-living amoebae as well as in macrophages (Hoffmann et al., 2014b; Boamah et al., 2017; Swart et al., 2018). This compartment restricts fusion with lysosomes, but communicates with the endosomal, secretory and retrograde vesicle trafficking pathways (Isberg et al., 2009; Hubber and Roy, 2010; Asrat et al., 2014; Personnic et al., 2016; Bärlocher et al., 2017b; Swart and Hilbi, 2020; Figure 1).

*D. discoideum* mutants were used to investigate the role of PI-metabolizing enzymes for intracellular growth of *L. pneumophila*. *D. discoideum* strains lacking PI3Ks (Weber et al., 2006; Peracino et al., 2010), PIKfyve (Buckley et al., 2019), Dd5P4/OCRL (Weber et al., 2009) or RpkA (Riyahi et al., 2011) are more permissive for *L. pneumophila* growth. In contrast, the deletion of PTEN does not affect intracellular growth, but reduces the uptake of *L. pneumophila* (Peracino et al., 2010), while PI3Ks are largely dispensable for bacterial uptake (Weber et al., 2006).

Decisive steps in the maturation of the LCV are a PI lipid conversion from the endosomal PtdIns(3)*P* to the secretory PtdIns(4)*P* (Weber et al., 2006, 2014; Steiner et al., 2018a; Swart and Hilbi, 2020), covalent and non-covalent subversion of small GTPases of the Arf, Rab, Ran, and Rap families (Rothmeier et al., 2013; Sherwood and Roy, 2013; Schmölders et al., 2017; Swart et al., 2020b), modulation of other trafficking factors such as the retromer coat complex (Finsel et al., 2013; Bärlocher et al., 2017a; Romano-Moreno et al., 2017; Yao et al., 2018), as well as the recruitment of tubular endoplasmic reticulum (ER) in *D. discoideum* and *A. castellanii* amoebae (Abu Kwaik, 1996; Lu and Clarke, 2005; Weber et al., 2014) and macrophages (Swanson and Isberg, 1995; Tilney et al., 2001; Figure 1). Proteomics of LCVs purified from infected *D. discoideum* revealed the presence of some of these factors on the pathogen vacuole (Urwyler et al., 2009; Hoffmann et al., 2014a; Schmölders et al., 2017). Interestingly, among the plethora of LCV host factors, we also identified dynamin superfamily proteins (DSPs), such as the fusion DSP Sey1/Atl3 and the scaffold DSP Gnbp (see below).

## Dynamin Superfamily Proteins: Mediators of Membrane Fission and Fusion

Cellular membranes are highly dynamic lipid bilayer structures that undergo constant remodeling. Among the numerous actors involved in the mechanisms underlying membrane remodeling, DSPs are well-studied large GTPases, which catalyze membrane fission and fusion events, and thus play key roles in endomembrane transport, vesicle biogenesis, organelle homeostasis, and cell division (Ramachandran and Schmid, 2018; Jimah and Hinshaw, 2019). In addition, DSPs play important roles in cell-autonomous immunity to fight pathogens, as well as in the organization and regulation of microtubules and the actin cytoskeleton (Shpetner and Vallee, 1989; Palmer et al., 2015; Zhang et al., 2020). DSPs are present in all eukaryotic cells, and even bacteria display dynamin-like proteins with functions in cytokinesis, toxin secretion and drug resistance (Bramkamp, 2012; Liu et al., 2018; Wang et al., 2019; Table 1).

**TABLE 1:** Eukaryotic dynamin superfamily proteins.

**Protein family**	**Representative DSP member**	***D. discoideum*** **ortholog**	**Function in** ***D. discoideum***
**Fission DSP** Dynamins	*H. sapiens* Dynamin-1 (Dnm1)	Not identified	Unknown
**Fission DSP** Dynamin-like	*H. sapiens* Dynamin-related protein-1 (Drp1)	Dynamin A (DymA) DDB_G0277849	Phagocytosis, cytokinesis
	*S. cerevisiae* Vacuolar protein sorting- associated protein-1 (Vps1)	Dynamin B (DymB) DDB_G0277851	Peroxisome scission
	Plant and algae Dynamin-related protein-5A (DRP5A)	Dynamin-like protein A, DlpA, DDB_G0268592	Cytokinesis, phagocytosis?
		Dynamin-like protein B, DlpB, DDB_G0285931	Cytokinesis
		Dynamin-like protein C, DlpC, DDB_G0271628	Cytokinesis?
**Fusion DSP** Atlastins	*H. sapiens* Atlastin-1-3 (Atl1-3)	Sey1/Atl3 DDB_G0279823	ER tubule fusion, exocytosis
**Fusion DSP** Mitofusin-like	*S. cerevisiae* Mitofusin-1 (Fzo1)	Similar to Fzo-1 DDB_G0287331	Unknown
**Membrane-independent scaffold DSP** Guanylate-binding proteins	*H. sapiens* GBP1-5	Gnbp DDB_G0281639	Unknown
**Membrane-independent scaffold DSP** Myxovirus resistance proteins	*H. sapiens* MxA-B	Not identified	Unknown
			

**FIGURE 2:**
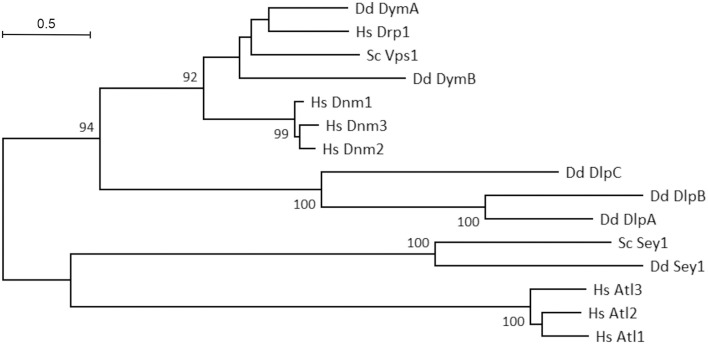
Phylogenetic tree of eukaryotic dynamin superfamily proteins. Evolutionary analyses were conducted using the MUSCLE algorithm for sequence alignment in the Molecular Evolutionary Genetics Analysis (MEGA) X software package (Kumar et al., 2018); the phylogenetic tree was built using the Maximum Likelihood method and Le-Gascuel model (Le and Gascuel, 2008). The tree is drawn to scale, with branch lengths corresponding to the number of substitutions per site. Numbers on the branches indicate bootstrap support for nodes from 500 bootstrap replicates (only values above 75% are shown). The scale bar represents the estimated evolutionary distance (number of amino acid substitutions per site).

The phylogenetic tree of eukaryotic DSPs reveals a number of interesting facts (Figure 2). The human dynamins (Dnm1–3) form a separate cluster and are related to *D. discoideum* DymA and DymB. Interestingly, these *D. discoideum* DSPs are even more closely related to human dynamin-related protein 1 (Drp1) and yeast (*S. cerevisiae*) vacuolar protein sorting-associated protein 1 (Vps1). The *D. discoideum* dynamin-like proteins (DlpA-C) form a separate cluster within the fission DSPs. Among the fusion DSPs, the human atlastins (Atl1-3) form a cluster, which is separated from *D. discoideum* and *S. cerevisiae* Sey1 (Atl3).

Several recent comprehensive reviews have covered DSP members, as well as the structural bases for their respective functions (Ramachandran and Schmid, 2018; Ford and Chappie, 2019; Jimah and Hinshaw, 2019; Kalia and Frost, 2019). Some general features common to all DSPs have emerged that we briefly present here. The main scope of this review is to focus on DSP members identified so far in *D. discoideum* and highlight their importance for cellular membrane dynamics and host-pathogen interactions.

## Structural and Functional Characteristics of Dynamin Superfamily Proteins

**FIGURE 3:**
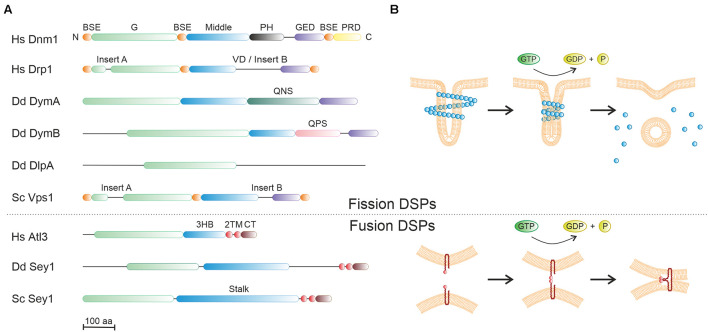
Molecular architecture and mode of action of dynamin superfamily proteins. **(A)** Domain organization of representative members of dynamin superfamily proteins (DSPs) from human, *D. discoideum* or *S. cerevisiae*. The large GTPase domain (green) is conserved, and the “stalk/middle” domains (blue) along with the BSE (orange) fold into a four- or three-helix bundle. BSE, bundle signaling element; G, GTPase domain; GED, GTPase effector domain; PH, pleckstrin homology domain; PRD, proline rich domain; 3HB, three-helix bundle; VD, variable domain; TM, transmembrane domain; CT, cytosolic domain; QNS, glutamine-asparagine-serine-rich domain; QPS, glutamine-proline-serine-rich domain. **(B)** The putative mode of action of fission DSPs (upper panel) and fusion DSPs (lower panel). The basic unit of fission and fusion DSPs is a dimer or a monomer, respectively. Fission DSPs (e.g., dynamin) are dimeric cytosolic proteins, which assemble into helical polymers around tubular target membranes. The assembly-stimulated GTPase activity likely promotes membrane constriction by shear forces, followed by DSP disassembly. In contrast, fusion DSPs (e.g., Sey1/atlastin) are monomeric membrane-anchored proteins, which dimerize only upon GTP binding, thus presumably tethering opposing membranes. GTPase activity triggers bending of the DSPs, which likely compresses the adjacent membrane lattices.

Dynamin was initially identified as a mechano-chemical GTPase, which bundles microtubules and catalyzes the constriction and scission of budding vesicles on the plasma membrane during clathrin-mediated endocytosis (Shpetner and Vallee, 1989; Ramachandran and Schmid, 2018; Jimah and Hinshaw, 2019). The signature structural feature of dynamin comprises a large GTPase (G) domain (∼280 amino acids) adjacent to a helical bundle stalk, which was subsequently identified in numerous proteins now regrouped under the generic name dynamin superfamily proteins (DSPs) (Ramachandran and Schmid, 2018; Jimah and Hinshaw, 2019; Figure 3A and Table 1). Some DSPs also contain supplementary domains providing diverse properties (e.g., protein/lipid interactions) (Ford and Chappie, 2019). Another common trait of DSPs is that these large GTPases are not regulated by guanine nucleotide exchange factors (GEFs) or GTPase-activating proteins (GAPs) (Binns et al., 2000; Narayanan et al., 2005), and their activity is greatly enhanced by nucleotide-induced dimerization (Praefcke and McMahon, 2004). Notably, the first identified dynamin and its homologs are now referred to as classical/modern dynamins, while other DSP members with diverse structural determinants (and functions) are collectively named dynamin-like or dynamin-related proteins, and most of them are regarded as ancestors of the modern DSP branch. DSPs can be functionally grouped in three main types, referred to as fission, fusion and membrane-independent scaffold DSPs (Ramachandran and Schmid, 2018).

**Fission DSPs** are cytosolic proteins that share among them a similar domain organization. Archetypes of this family include classical dynamins and some dynamin-like proteins (Ramachandran and Schmid, 2018; Table 1). Three isoforms of classical dynamin exist in vertebrates. While dynamin-1 (Dnm1) is exclusively present in neurons, dynamin-2 (Dnm2) is ubiquitously produced, and dynamin-3 (Dnm3) is mainly present in neurons and testes. These dynamins harbor five domains: a catalytic N-terminal G domain, a bundle signaling element (BSE), a four alpha-helix stalk, a phospholipid-interacting pleckstrin homology (PH) domain, and a C-terminal proline/arginine-rich domain (PRD) that interacts with SH3 domain-containing proteins (Figure 3A).

Extensive structural studies have elucidated the overall architecture of dynamin in solution and bound to membranes, providing insights into the mechanism of membrane scission (Chappie et al., 2011; Faelber et al., 2011; Ford et al., 2011; Figure 3B, upper panel). Accordingly, dynamins are obligate homodimers, wherein the stalk of each monomer interacts in a criss-cross fashion. This dimeric building block can similarly assemble via two additional stalk interfaces into a tetramer. Further assembly is prevented by a regulatory interaction between the PH domain and the stalk domains. Upon recognition of the membrane PI lipid phosphatidylinositol 4,5-diphosphate (PtdIns(4,5)*P*_2_) by the PH domain, this inhibition is removed, and dynamin switches from a closed to an open conformation. The structural change enables the self-assembly into helical polymers on target membranes and stimulates the GTPase activity. This will eventually drive membrane fission and subsequent disassembly of helical polymers (Figure 3B, upper panel), though the actual fission mechanism is still controversial (Ford and Chappie, 2019). Remarkably, a newly developed single-particle orientation and rotational tracking technique has recently revealed that dynamin drives a strong rotational twist of clathrin-coated vesicles, which might assist the vesicle scission step (Cheng et al., 2021).

A representative fission DSP that belongs to the dynamin-like group is mammalian Drp1, which is a major mitochondrial fission GTPase and also contributes to peroxisome division (Kraus et al., 2021; Figure 3A). In Drp1, the PH and PRD domains of classical dynamins are replaced by an unstructured region known as the variable domain (VD), which is responsible for the recruitment to the mitochondrial outer membrane, but can also inhibit Drp1 self-assembly in the absence of membrane interactions. In a GTP-dependent manner, Drp1 forms helical oligomers bound to the outer mitochondrial membrane inducing membrane constriction and severing (Kraus and Ryan, 2017). Drp1 also promotes the intracellular replication of *L. pneumophila* in LCVs, likely due to its role in catalyzing microtubule-dependent mitochondrial fission and its role in cellular bioenergetics (Escoll et al., 2017).

Besides their membrane fission activity, dynamin and the dynamin-like group of DSPs exert additional functions. Notably, classical dynamins interact with microtubules and the actin cytoskeleton and regulate the formation and the function of these cytoskeletal structures (Menon and Schafer, 2013). In addition to its actin-binding capacities (Ji et al., 2015), mammalian Drp1 also physically interacts with specific mitochondrial membrane adaptors. In turn, the adaptors affect the remodeling of the inner mitochondrial membrane mediated by the DSP Opa1, leading to the release of cytochrome c during intrinsic apoptosis (Otera et al., 2016).

**FIGURE 4:**
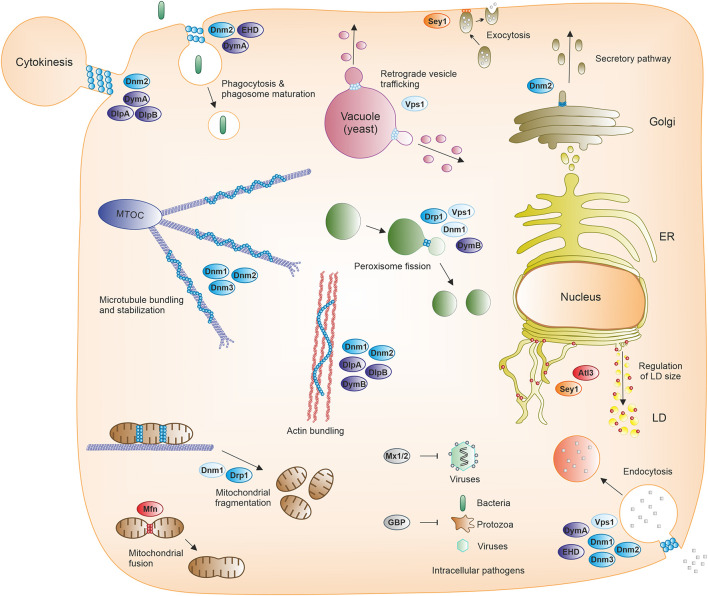
Cellular processes mediated by dynamin superfamily proteins. Fission, fusion, and membrane-independent scaffold DSPs participate in various cellular processes, including endomembrane transport (such as phagocytosis, macropinocytosis, endocytosis, secretory and retrograde trafficking, or autophagy), organelle and cytoskeleton homeostasis, cytokinesis, and immunity against viral, bacterial and protozoan pathogens. ER, endoplasmic reticulum; GBP, guanylate-binding protein; LD, lipid droplet; MTOC, microtubule-organizing center; Mx1/2, Myxovirus resistance protein 1/2.

Vps1 is a conserved fungal DSP, which shares many structural features with Dnm1, such as the G, BSE, middle and GED domains (Varlakhanova et al., 2018; Figure 3A and Table 1). In addition, Vps1 contains two inserts, Insert A and Insert B, the function of which is unknown. Vps1 has been implicated in both membrane fission and fusion processes (Peters et al., 2004), as well as in peroxisome homeostasis, endocytosis (Smaczynska-De Rooji et al., 2010; Varlakhanova et al., 2018), and retrograde trafficking originating from endosomes or the vacuole (Chi et al., 2014; Arlt et al., 2015; Figure 4).

**Fusion DSPs** are preferentially inserted into or anchored to membranes and exist as monomers. Atlastins and mitofusins are typical members of this DSP class, which share a similar domain organization and fusogenic mechanism (Moon and Jun, 2020), and are involved in homotypic ER tubule fusion or mitochondrial outer membrane fusion, respectively (Table 1). Moreover, atlastins also promote lipid droplet biogenesis and size regulation (Klemm et al., 2013) (Figure 4). Atlastins are found in all eukaryotic cells and composed of a cytosolic N-terminal G domain, a three-helix bundle (3HB) region (analogous to the middle/stalk/BSE domains of other DSPs), two transmembrane (TM) segments (forming intramembrane hairpin loops) (Betancourt-Solis et al., 2018) and a C-terminal tail (Figure 3A). GTP binding induces atlastin homodimerization either on the same (*cis*) or opposing membranes (*trans*), whereas GTP hydrolysis seems to promote the disassembly into monomers (Figure 3B, lower panel). The TM domains are required for fusion while the cytoplasmic tail mediates membrane tethering. In addition to their ER fusogenic function, atlastins are involved in an impressive number of processes including vesicle packaging, endocytic trafficking, lipid droplet biogenesis, ER-phagy, microtubule dynamics, calcium signaling and protein homeostasis (Lü et al., 2020), as well as bacterial infection (see below).

Mitofusins in mammals (Fzo1 in yeast) contain an N-terminal G domain, a heptad repeat region (HR1), two TM regions, and a second heptad repeat region (HR2). Upon GTP hydrolysis, mitofusins from two different membranes dimerize via their G domain, subsequently inducing conformation changes that bring the membranes closer together and enable their fusion. Whereas the HR2 domains contribute to dimerization of mitofusins on opposing membranes by forming antiparallel dimers, the HR1 domain facilitates membrane fusion by perturbing local membrane structures in its vicinity (Gao and Hu, 2021).

**Membrane-independent scaffold DSPs** comprise two main representative members, guanylate-binding proteins (GBPs) and Myxovirus resistance (Mx) proteins, the closest DSP homologs of which are atlastins (Table 1). GBPs are induced by interferon-γ (IFN-γ) as immunity-related GTPases in vertebrates and are involved in the cellular immune responses against bacterial, viral, and protozoan pathogens (Ngo and Man, 2017; Tretina et al., 2019; Figure 4). These DSPs also display antitumor and antiproliferative properties, as well as actin remodeling activities. The architecture of GBPs consists of an N-terminal G domain followed by an extended C-terminal helical domain. Three of the seven human GBP (GBP1, –2, –5) possess a CaaX box at the C-terminus required for post-translational isoprenylation, which promotes the association with membranes and/or pathogens. GTP binding promotes the self-assembly of GBPs into homodimers but also into supramolecular structures (several thousand monomers). These nanostructures can deposit on pathogen-associated membranes and serve as mechano-enzymes fatally disrupting membranes from pathogens and/or vacuoles containing pathogens in host cells. The nanostructures can also function as cellular sensory platforms for the detection of pathogens and the subsequent induction of several defense mechanisms, including inflammasome-driven responses (Tretina et al., 2019).

The production of Mx proteins also strictly depends on interferon signaling and contributes to the innate immunity against diverse RNA viruses (Haller et al., 2015; Figure 4). Two Mx proteins exist in humans. Mx1 (MxA) shows a wide antiviral activity against RNA and DNA viruses. In contrast, Mx2 (MxB) activity is restricted to only a few viruses including HIV-1. Similarly to classical dynamin, Mx proteins harbor G, BSE and stalk domains, but the PH and PRD domains are missing. Instead, a 40 amino acid loop (L4 loop) replaces the PH domain and mediates membrane lipid and viral target interactions. The antiviral activity depends on the GTP-dependent multimeric assembly of the Mx proteins. Mx oligomers form ring-like structures with the G domains directed to the outer side and the stalks located on the opposite inner side. These Mx rings might inhibit different steps of viral replication cycles occurring in the cytosol and the nucleus, although the actual antiviral mechanism is still not clearly established.

Taken together, DSPs participate in various cellular processes, including endomembrane transport (such as phagocytosis, macropinocytosis, endocytosis, secretory pathway, retrograde trafficking, autophagy), organelle and cytoskeleton homeostasis, cytokinesis, and immunity against viral, bacterial and protozoan pathogens (Figure 4).

## Dynamin Superfamily Proteins in *D. discoideum*

In *D. discoideum* five dynamin-like proteins, DymA (gene ID: DDB_G0277849), DymB (DDB_G0277851), DlpA (DDB_G0268592), DlpB (DDB_G0285931) and DlpC (DDB_G0271628), as well as the fusion DSP Sey1/Atl3 (DDB_G0279823) have been described (Figure 3A and Table 1). Moreover, BLAST searches have revealed putative orthologs of a mammalian GBP (DDB_G0281639) and a yeast Fzo1/mitofusin-like DSP (DDB_G0287331). Since there are only few DSPs in *D. discoideum* compared to vertebrates, we discuss here each individual member, rather than adopting a classification based on their functions (e.g., phagocytosis, organelle morphogenesis, cytokinesis) (Figure 4).

## Dynamin A Promotes Endocytosis and Phagosome Maturation

Dynamin A (DymA) is most similar to the mammalian fission dynamin-1-like protein (Drp1) and is classified as a dynamin-related protein (DRP) or ancient DSP. The 3D structure of its G domain was the first to be solved among DSPs (Niemann et al., 2001). One characteristic feature is the replacement of the PH and PRD domains found in classical dynamin by a segment, which is rich in asparagine, glutamine and serine residues, inserted between the middle domain (also known as three-helix bundle domain) and the C-terminal GED (Figure 3A). This domain might contribute to the reported interaction of DymA with membrane lipids (Klockow et al., 2002) or the regulation of G domain dimerization.

DymA was first identified on post-lysosomes, mitochondria and Golgi compartments (Wienke et al., 1999). Later studies have additionally revealed its recruitment to phagosomes containing latex beads during the early phase of the phagocytic process but also to some extent at late/final stages (Gopaldass et al., 2012; Figure 4). DymA was also shown to localize to the furrow of dividing cells during the entire cytokinesis process (Masud Rana et al., 2013; Figure 4). However, recent live cell imaging has revealed a more specific localization restricted to the intercellular bridge only during the final separation phase of dividing cells (Fujimoto et al., 2019).

As implied by these numerous cellular localizations, deletion of DymA results in pleiotropic effects comprising alterations in mitochondrial morphology and functions, impairment of cytokinesis and endosomal morphology (accumulation of membrane tubules in proximity to nuclei and plasma membrane), as well as a defect in fluid-phase uptake and increased phagocytic rates (Wienke et al., 1999). Whether these multiple defects reflect direct DymA functions or are indirect consequences of the DymA deletion remains unclear. For instance, the defective mitochondrial morphology in *dymA* null cells is not associated with any mitochondrial dynamics and fission/fusion defects (Schimmel et al., 2012), but instead is likely due to other impaired DymA-dependent cellular processes. Moreover, *dymA* null cells show an aberrant localization of myosin II and disorganized actin filaments at the furrow of dividing cells (Masud Rana et al., 2013). DymA was also shown to interact with the actin-binding protein Abp1 (Dieckmann et al., 2010) and could thus regulate actin assembly at the furrow rather than or in addition to exhibiting membrane fission activity at this site. Instead, the latter function might rely on DlpA and DlpB, two other DSP more abundantly present at the cleavage furrow (Fujimoto et al., 2019) (see below).

Phagosomes are well described to undergo several maturation steps involving membrane vesicle fusion and fission events (Kinchen and Ravichandran, 2008). In the absence of DymA, phagosome acidification is impaired, a clear indication of its direct role in the phagosomal maturation process (Gopaldass et al., 2012). F-actin binding to early phagosomes is also compromised in *dymA* null cells, in agreement with functions of DymA independent of vesicle scission. Accordingly, DymA also interacts with the C-terminal Eps15-homology-domain (EHD) protein (Gueho et al., 2016), which seems to further extend the functional spectrum of DymA. EHDs are ATPase mechano-enzymes related to the dynamin superfamily, implicated in membrane scission and the regulation of endocytic events (Naslavsky and Caplan, 2011). In addition to membrane remodeling functions, *D. discoideum* EHD interacts with other binding partners (Gueho et al., 2016), and thus, EHD might adopt scaffolding functions together with DymA.

Moreover, a possible interplay between DymA and other DSP members cannot be excluded. DSPs can form hetero-multimers with other DSP members such as DlpA (see below), and these cooperative arrangements might support new functions. Hence, DlpA (independently of DlpB) was shown to accumulate at the phagocytic cups and on nascent phagosomes containing bacteria (Fujimoto et al., 2019). Hypothetically, DlpA might transiently form heterodimers with DymA and/or EHD on newly formed phagosomes, and these complexes might contribute to the phagosomal maturation process (Dieckmann et al., 2010; Gueho et al., 2016).

## Dynamin B Regulates Membrane Fission and Actin Dynamics

Dynamin B (DymB) is classified as a dynamin-like protein and shares the same domain organization as DymA, including the substitution of the PH and PRD domains by an unstructured domain rich in glutamine, proline, and serine residues (Rai et al., 2011; Figure 3A). In addition, DymB possess a particular N-terminal sequence (136 amino acids) responsible for its localization to mitochondria (Rai et al., 2013). Such an N-terminal mitochondrial targeting sequence (MTS) is notably also observed in the human dynamin-like GTPase Opa1 implicated in mitochondrial fusion. However, a phylogenetic analysis indicates that DymB is more closely related to yeast Vps1, a dynamin-like protein involved in endocytosis, vacuolar trafficking and peroxisomal division (Miyagishima et al., 2008; Figure 2). After cleavage of the N-terminal peptide by mitochondrial proteases, the mature DymB protein still localizes to the outside of the outer mitochondrial membrane, in contrast to Opa1, which associates with the outer surface of the inner mitochondrial membrane.

DymB localization was initially determined in DymB-GFP expressing cells, revealing a predominant distribution to mitochondria and a minor presence on other organelles and the plasma membrane (Rai et al., 2011). However, endogenous DymB detected with a specific antibody appears to localize mainly to the cytosol (Masud Rana et al., 2013). Thus, DymB might be weakly or transiently associated with mitochondria, and might also function in various other cellular compartments (Figure 4). Accordingly, DymB-depleted cells do not present any mitochondrial morphological and functional defects and are fully competent for mitochondrial fission (Rai et al., 2011; Schimmel et al., 2012). Instead, the absence of DymB results in multiple defects including increased peroxisome size and impaired peroxisome function, increased tubulation of the contractile vacuole system and decreased contractile activity of large cisternae, as well as dysregulation of F-actin formation and distribution (Rai et al., 2011). In conclusion, DymB adopts membrane scission functions at various cell locations and regulates actin assembly as well as actin-dependent processes. It is unknown whether the transient localization of DymB to mitochondria contributes to the regulation of the amount of cytosolic enzyme by controlling the rate limiting proteolysis step. Alternatively, mitochondria, through their frequent interactions with other organelles at membrane contact sites (MCS), might support DymB delivery to specific sites.

## The Dynamin-Like Proteins DlpA, DlpB and DlpC Regulate Cytokinesis

In addition to DymA and DymB, *D. discoideum* produces three dynamin-like proteins (DlpA, DlpB, and DlpC), which harbor only a DSP-characteristic G domain near their N-terminus without any other known domains (InterProScan and Prosite analyses) (Figure 3A). Their primary sequences are quite similar (sequence identity between DlpA and DlpB > 36%; DlpA or DlpB and DlpC > 27%). Phylogenetic analyses indicate that these DSPs belong to the monophyly comprising plant and algae DRP5A proteins involved in cytokinesis, and they display a distant relationship with chloroplast division DRP5B proteins (Miyagishima et al., 2008).

A seminal study first revealed the implication of DlpA, DlpB, and DlpC in cytokinesis, as individual gene deletions resulted in multinucleated enlarged cells, and DlpA was present on the cleavage furrow (Miyagishima et al., 2008). This observation was partially confirmed for DlpA and DlpB, but not for DlpC, whose inactivation had no impact on cytokinesis (Masud Rana et al., 2013). To clarify this discrepancy, the function of DlpC in cytokinesis needs to be readdressed with an independently generated *dlpC* knockout strain. Indeed, genetic compensation in response to gene knockout is a widespread phenomenon that could explain such phenotypic differences (El-Brolosy and Stainier, 2017). For instance, upregulation of DlpA and DlpB in the existing *dlpC* mutant might compensate the cytokinesis defect in this case.

Interestingly, DlpA and DlpB have to form hetero-oligomers to be recruited to the cleavage furrow (Fujimoto et al., 2019). As described for *dymA* null cells, deletion of either DlpA or DlpB induced the disorganization of the actomyosin cytoskeleton at the furrow region (Masud Rana et al., 2013; Fujimoto et al., 2019). Both proteins were shown to associate with the actin cytoskeleton and stabilize actin filaments. In the current working model, DlpA, DlpB, and DymA are proposed to cooperate in the cytokinesis process. Accordingly, DlpA and DlpB would stabilize the actin filament in the contracting ring, whereas DymA would be recruited to the intercellular bridge only during the final separation step and scission of the dividing cells (Fujimoto et al., 2019).

A puzzling observation is the accumulation of DlpA without DlpB at the phagocytic cup formed during uptake of bacteria (Fujimoto et al., 2019). The molecular basis for this transient and selective DlpA recruitment to phagosomal membranes is unknown. DlpA recruitment might involve the putative participation of DlpA-binding partners specifically associated with nascent phagosomes and/or the possible cooperation with other DSP members. Moreover, the function of DlpA on the phagosomal cup has yet to be assessed. Similarly to its role in cytokinesis, DlpA might presumably regulate the actin cytoskeleton dynamics during phagocytic cup formation, although a membrane scission function cannot be excluded. Notably, such a dynamin-actin cross-talk has been described for Dnm2 in macrophages during both phagosome formation and closure, preceding the function of Dnm2 in phagosome scission (Marie-Anais et al., 2016). Further detailed analysis of phagocytosis in *dlpA* null cells should reveal new insights into DlpA functions.

## *D. discoideum* Sey1 Promotes Endoplasmic Reticulum Dynamics and *Legionella* Growth

Sey1/Atl3 is the only atlastin ortholog present in *D. discoideum*, and it shares the same domain organization as the mammalian atlastins described above (Steiner et al., 2017; Figure 3A). Sey1 localizes to interconnected ER membranes and ER tubules as well as to perinuclear ER membranes. To determine its function, we have recently generated and characterized a *sey1* deletion mutant strain (Δ*sey1*) (Hüsler et al., 2021). Cells lacking Sey1 show pleiotropic defects, including aberrant ER morphology and ER tubules dynamics, defective lysosomal enzymes exocytosis and intracellular proteolysis, impaired cell motility, and inability to cope with prolonged ER stress. Remarkably, all these defects were complemented by plasmid-borne wild-type Sey1, but not by a catalytically-inactive Sey1 variant (Sey1_K154A), indicating that Sey1 GTPase-dependent functions are important for the normal course of these cellular processes.

It is unclear at present whether the defects observed in Δ*sey1* cells are a direct consequence of the absence of Sey1 GTPase-dependent function(s), or whether these defects are due to the altered ER morphology in the mutant cells. For instance, the tubular ER network forms abundant MCS with organelles and the plasma membrane to support exchanges between organelles (e.g., lipids, Ca^2+^), and it participates in various other cellular processes (Phillips and Voeltz, 2016). Hence, the altered ER network in Δ*sey1* cells might impede the formation, location or function of these ER-MCS, and consequently this would result in the dysfunction of multiple MCS-dependent cellular processes. Further studies will be required to explore this hypothesis and the role of Sey1 in different functions reported for atlastins in other organisms (e.g., ER-phagy, lipid droplet biogenesis).

Intriguingly, the intracellular replication of *L. pneumophila* has recently been shown to be regulated by Sey1/Atl3 (Steiner et al., 2017, 2018b; Figure 1). Sey1 was found to localize to early LCVs, and over production of the DSP promoted intracellular growth of *L. pneumophila*. Production of a catalytically inactive, dominant-negative Sey1_K154A mutant protein, or Atl3 depletion, restricted pathogen replication and impaired LCV maturation. An ultrastructural analysis by electron microscopy confirmed that dominant-negative Sey1 compromises ER accumulation on LCVs. Of note, Sey1 was not required for the formation of the PtdIns(4)*P*-positive LCVs and initial ER recruitment, but for the DSP-catalyzed pathogen vacuole expansion. Moreover, *D. discoideum* lacking the *sey1* gene was found to be less permissive for intracellular *L. pneumophila* replication (Hüsler et al., 2021). LCV formation was impaired in this mutant strain, since the ER was less efficiently recruited to the pathogen vacuole, and LCV expansion was retarded at early stages of infection. Taken together, the DSP Sey1/Atl3 controls circumferential ER remodeling during LCV maturation and intracellular replication of *L. pneumophila*. The molecular mechanism of how Sey1/Atl3 promotes LCV expansion and *L. pneumophila* growth has not been elucidated, but might involve a more efficient delivery of ER to LCV-ER contact sites and lipid exchange among the compartments.

## The Guanylate-Binding Protein Gnbp Is Possibly Implicated in Pathogen-Host Cell Interactions

As mentioned above, mammalian GBPs are DSPs regulating inflammasome-dependent responses to bacterial infection (Tretina et al., 2019). *D. discoideum* harbors a single GBP homolog encoded by the *gnbp* gene (Dunn et al., 2017), which might adopt similar immune functions in the amoeba (Figure 4). Protein domain prediction (InterProScan, TMHMM Server v. 2.0) revealed the presence of an N-terminal signal peptide, a conserved G domain, and a C-terminal TM domain. The helical part of human GBP following the G domain is replaced by a long protein sequence in *D. discoideum* with no predicted structural motif. This predicted domain organization suggests that *D. discoideum* GBP might be constantly inserted into membranes independently of GTP binding, as observed in atlastins and in contrast to GBPs in other species. Similarly to atlastin, *D. discoideum* GBP might thus display membrane tethering and/or fusion properties. Interestingly, tethering functions have been previously described for Gbp1 (Shydlovskyi et al., 2017). In addition, *D. discoideum* GBP has been described as a putative binding partner of Sec7, a GEF specific for the small GTPase ARF (ADP-ribosylation factor), implicated in vesicular trafficking (Müller et al., 2013). *D. discoideum* GBP is also present in the proteome of macropinosomes, and therefore, this protein is likely to be involved in vesicular trafficking along the endocytic pathway (Journet et al., 2012). Whether *D. discoideum* GBP also plays a role in cell-autonomous immunity similarly to other GBPs is unknown.

Intriguingly, Gnbp was also identified in the proteome of LCVs purified from *L. pneumophila*-infected *D. discoideum* (Hoffmann et al., 2014a; Figure 1). This finding is in agreement with the notion that the *D. discoideum* GBP is implicated in the recognition of and/or defense against intracellular pathogens. However, the function of this scaffold DSP on the LCV or in the context of an *L. pneumophila* infection remains to be elucidated.

## Conclusion and Outlook

The soil amoeba *D. discoideum* is a versatile model organism to study vesicle trafficking, motility and migration, cell division, as well as host cell-pathogen interactions. Large GTPases of the dynamin superfamily play crucial roles for membrane dynamics, vesicle trafficking, organelle homeostasis, cytokinesis and cell-autonomous immunity. Insights into the cellular localization, function and structure of DSPs have soared over the last years. *D. discoideum* produces at least eight DSPs, which are involved in membrane fission and fusion events, as well as possibly in cell-autonomous immunity. Moreover, the amoebae produce one ortholog of mammalian atlastins, Sey1, which is implicated in ER dynamics and intracellular growth of the opportunistic pathogen *L. pneumophila*. Amoebae deleted for Sey1 show pleiotropic defects, including aberrant ER morphology and dynamics, as well as impaired pathogen vacuole formation and replication of *L. pneumophila*. We propose to harness the wide array of biochemical, genetic and cell biological tools available for *D. discoideum* to further exploit the functions of DSPs in cellular processes and upon infection with pathogens.

## Author Contributions

FL and HH wrote the manuscript. AK and DH prepared the figures and edited the manuscript. All authors contributed to the article and approved the submitted version.

## Conflict of Interest

The authors declare that the research was conducted in the absence of any commercial or financial relationships that could be construed as a potential conflict of interest.

## Publisher’s Note

All claims expressed in this article are solely those of the authors and do not necessarily represent those of their affiliated organizations, or those of the publisher, the editors and the reviewers. Any product that may be evaluated in this article, or claim that may be made by its manufacturer, is not guaranteed or endorsed by the publisher.

## Footnotes

1
http://dictybase.org/

## Abbreviations

GPCR: G protein coupled receptor
Icm/Dot: intracellular multiplication/defective organelle trafficking
LCV: *Legionella*-containing vacuole
PI: phosphoinositide
PtdIns: phosphatidylinositol
T4SS: type IV secretion system.
